# I Am vs. We Are: How Biospheric Values and Environmental Identity of Individuals and Groups Can Influence Pro-environmental Behaviour

**DOI:** 10.3389/fpsyg.2021.618956

**Published:** 2021-02-18

**Authors:** Xiao Wang, Ellen Van der Werff, Thijs Bouman, Marie K. Harder, Linda Steg

**Affiliations:** ^1^Department of Psychology, Faculty of Behavioural and Social Sciences, Groningen University, Groningen, Netherlands; ^2^Department of Environmental Science and Engineering, Fudan University, Shanghai, China; ^3^School of Computing, Engineering and Mathematics, University of Brighton, Brighton, United Kingdom

**Keywords:** biospheric values, environmental identity, personal and group approach, pro-environmental behaviour, sustainability, cross-cultural study

## Abstract

Most research in environmental psychology is conducted in individualistic countries and focuses on factors pertaining to individuals. It is yet unclear whether these findings also apply to more collectivistic countries, in which group factors might play a prominent role. In the current paper, we test the individual-focused value–identity–behaviour pathway, in which personal biospheric values relate to pro-environmental actions via environmental self-identity, in an individualistic and a collectivistic country. Furthermore, we test in both countries whether a new group-focused pathway also exists, in which group values relate to pro-environmental behaviour via environmental group identity, particularly in collectivistic countries. Questionnaire studies were conducted among Dutch (*N* = 161) and Chinese (*N* = 168) students. Our results indicated that personal biospheric values, mostly via environmental self-identity, predict pro-environmental behaviour in both countries. We also found initial support for our newly proposed value–identity–behaviour pathway at the group level, particularly in China. Yet, in both countries, the association between group-level variables and pro-environmental behaviour was weaker than for personal-level variables, and partly overlapped with personal-level variables. Our findings show the relevance of personal- and group-level factors in understanding pro-environmental behaviour in both individualistic and collectivistic countries, which has strong theoretical and practical implications, particularly for developing international strategies to promote pro-environmental actions across the world.

## Introduction and Hypothesis

Behaviour change is crucial to sustainability, especially when it comes to the mitigation of human-caused ongoing environmental problems (e.g., climate change, pollution) (Fischer et al., 2012; Goudie, 2013; UNFCCC, 2015; IPCC, 2018). To move towards a sustainable lifestyle, individuals need to engage in various pro-environmental behaviour urgently: behaviour that minimises the negative impact of one's actions on nature and the environment (Kollmuss and Agyeman, 2002; IPCC, 2018). Despite continuous efforts to promote pro-environmental behaviour, more actions are needed to reach international climate targets (IPCC, 2018). Interventions would be more efficient and effective if they target key antecedents of the desired sustainable behaviour (Steg, 2017). Thus, it is crucial to acquire a profound understanding of factors that may underlie and promote pro-environmental behaviour all over the world.

It has been theorised that pro-environmental behaviour is rooted in biospheric values, which reflect the importance people attach to caring about nature and the environment (Stern and Dietz, 1994; De Groot and Steg, 2010). Whereas, both individuals and groups are believed to endorse values, research so far mainly focused on the influence of personal values on pro-environmental behaviour and showed that personal biospheric values often indirectly predict a range of pro-environmental behaviour (Schultz and Zelezny, 1999; De Groot and Steg, 2009; Van der Werff et al., 2013a). Nevertheless, perceived group values may promote pro-environmental behaviour as well (Bouman and Steg, 2019, 2020), and this might be particularly the case in collectivistic cultures where people are more likely to act in line with the group interests (Triandis, 1988). Accordingly, the current paper aims to test how personal biospheric values and perceived group values relate to pro-environmental behaviour.

Specifically, in the present study, we aim to replicate the well-established personal-level pathway wherein personal biospheric values motivate pro-environmental behaviour via strengthening individuals' environmental self-identity (i.e., the degree to which individuals see themselves as environmentally friendly) and investigate whether this pathway can be extended to the group level, that is, whether perceived group biospheric values can motivate pro-environmental behaviour via environmental group identity (i.e., the degree to which the group is seen as environmentally-friendly). Importantly, we test the relevance and the robustness of the personal and group pathways in predicting pro-environmental behaviour in an individualistic country (i.e., the Netherlands) and a collectivistic country (i.e., China).

Personal values are stable, desirable and trans-situational goals that guide individual attitudes, evaluations and behaviour (Schwartz, 1992). Individuals endorse all values to some degree, but differ in how much they endorse and prioritise each value. The more someone endorses and prioritises a value, the more decisive this value will be for this person's attitudes, evaluations and behaviour. When focusing on the environmental domain, previous research identified four types of personal values that are most clearly related to pro-environmental behaviour, namely, altruistic, egoistic, hedonic, and biospheric values (De Groot and Steg, 2007b; Steg et al., 2014). Biospheric and altruistic values advocate benefits for the environment or others, respectively. The more individuals endorse biospheric and altruistic values, the more they tend to act pro-environmentally. In contrast, egoistic and hedonic values advocate self-interest and personal comfort. The more individuals endorse egoistic and hedonic values, the more reluctant they generally are to act pro-environmentally, mostly because pro-environmental behaviour can be financially costly, effortful or uncomfortable (De Groot and Steg, 2009; Steg et al., 2014; Jans et al., 2018; Bouman and Steg, 2019). Personal biospheric values appear particularly strong and robust predictors of pro-environmental attitudes, intentions and behaviour (Ojea and Loureiro, 2007; De Groot and Steg, 2009; Van der Werff and Steg, 2016; Bouman et al., 2018), which is why we focus on biospheric values in the current paper.

Personal biospheric values often influence pro-environmental behaviour indirectly, and one crucial mediator is someone's environmental self-identity (Van der Werff et al., 2013a). Self-identity is the label that one uses to describe oneself (Cook et al., 2002). Accordingly, environmental self-identity is defined as the extent to which individuals see themselves as someone who acts in an environmentally friendly way (Van der Werff et al., 2013a). The stronger one's environmental self-identity is, the more likely people are to engage in a wide range of pro-environmental behaviour (Cornelissen et al., 2008; Whitmarsh and O'Neill, 2010) because people are motivated to be consistent and act in line with how they see themselves (Van der Werff et al., 2013b). When people strongly care about nature and the environment—that is, when they strongly endorse biospheric values—they are more likely to see themselves as an environmentally friendly person; in turn, the more people consider themselves as environmentally friendly, the more likely they behave in pro-environmental ways (Gatersleben et al., 2012; Van der Werff et al., 2013a).

In addition to personal factors, such as personal biospheric values and environmental self-identity, group factors might play a role in predicting pro-environmental behaviour (Hornsey et al., 2006; Bouman and Steg, 2019, 2020). Yet, the role of group values and environmental group identity are less studied. Therefore, we investigate whether, in parallel to the personal-level pathway, a similar group-level pathway might exist and could also predict pro-environmental behaviour.

Groups generally guide what kind of beliefs and behaviour are appropriate for members (Tajfel, 1974; Feldman, 1984; Terry and Hogg, 1996; Hornsey, 2008). Accordingly, what people think is important to (i.e., perceived group values) and how they characterise (i.e., environmental group identity) their group may influence their beliefs and behaviour (Jans et al., 2018; Bouman and Steg, 2019, 2020). Extending this research, and similar to the personal-level pathway, we propose that perceived group biospheric values (i.e., the extent to which individuals think their group values the environment) may promote pro-environmental behaviour among group members via strengthening an environmental group identity.

Studies so far have shown that perceived group values may influence group members' behaviour, including pro-environmental behaviour (Hanel et al., 2018; Sanderson et al., 2019; Bouman et al., 2020a; Fielding et al., 2020). For example, organisational values have been proven to encourage employees' pro-environmental product purchasing behaviour, particularly when employees identify with the company's environmental concern (Cambra-Fierro et al., 2008). However, these studies mostly focused on general national values, organisational values or group political values, which did not emphasise the group environmental values. A few very recent studies began to discuss how perceived group's biospheric values may be critical in promoting individuals' pro-environmental behaviour as well (Jans et al., 2018; Bouman and Steg, 2019, 2020; Bouman et al., 2020a), yet they did not empirically study the process through which these values may translate into action. We will address this gap in the literature and examine the role of group biospheric values in motivating pro-environmental behaviour.

We propose that perceived group biospheric values may similarly relate to pro-environmental behaviour as personal values, but via the environmental identity at the group level. That is, the more people think their group cares about the environment, the more likely they are to see the group as a group that acts environmentally friendly. This stronger environmental group identity may, in turn, promote pro-environmental behaviour. Group identities have been found to influence pro-environmental behaviour (Fielding and Hornsey, 2016). However, most group identities studied before were not directly linked to the environment. For instance, a left-wing political identity was found to influence attitudes towards climate change policy (Unsworth and Fielding, 2014). Yet, a few studies investigated constructs similar to environmental group identity, such as “green consumer,” suggesting that such group identities are promoting pro-environmental behaviour (Moisander, 2000). Nevertheless, to our knowledge, there has not been a study linking group environmental values and group environmental identity together to reveal their relationship with pro-environmental behaviour. Thus, we will extend the current knowledge by investigating the relationship between group biospheric values, environmental group identity and pro-environmental behaviour. We will test if this pathway influences environmental behaviour in addition to the personal pathway, where the association between personal biospheric values and pro-environmental behaviour was mediated by environmental self-identity.

We conduct the study in the Netherlands and China to test whether we could identify the personal and group pathway in two culturally different countries. Previous studies on the personal pathway have been conducted in European countries, Australia or US (e.g., Gatersleben et al., 2012; Van der Werff et al., 2013a; Balunde et al., 2019). Therefore, our study aims to test the robustness and generalisability of the personal pathway with participants from an East Asian country. In addition, we investigate whether our novel group pathway exists in both countries.

It is to be noted that the main purpose of testing both pathways with diverse samples is to test the generalisability of findings, rather than testing for cultural difference, which would arguably require more national representative samples. Yet, we do *explore* whether the influence of both pathways may differ across countries. Cross-cultural studies suggest that China's culture is collective-oriented, while the Netherlands is individual-oriented (Hall, 1977; Hofstede, 2001). Chinese people are found to consider themselves more strongly as part of a larger whole and often prioritise the group's needs over the individual's needs than those in the individualistic counties, such as people in the Netherlands (Triandis, 1989). When the group and individual interests conflict, people from collectivistic cultures more often give priority to the group interests than people in individualistic cultures do (Brewer and Chen, 2007). Accordingly, perceived group values and group identity may be more influential in a collectivistic culture than in an individualistic culture, and the personal values and identity may be more influential in an individualistic culture than a collectivistic culture^1^.

**Figure 1:**
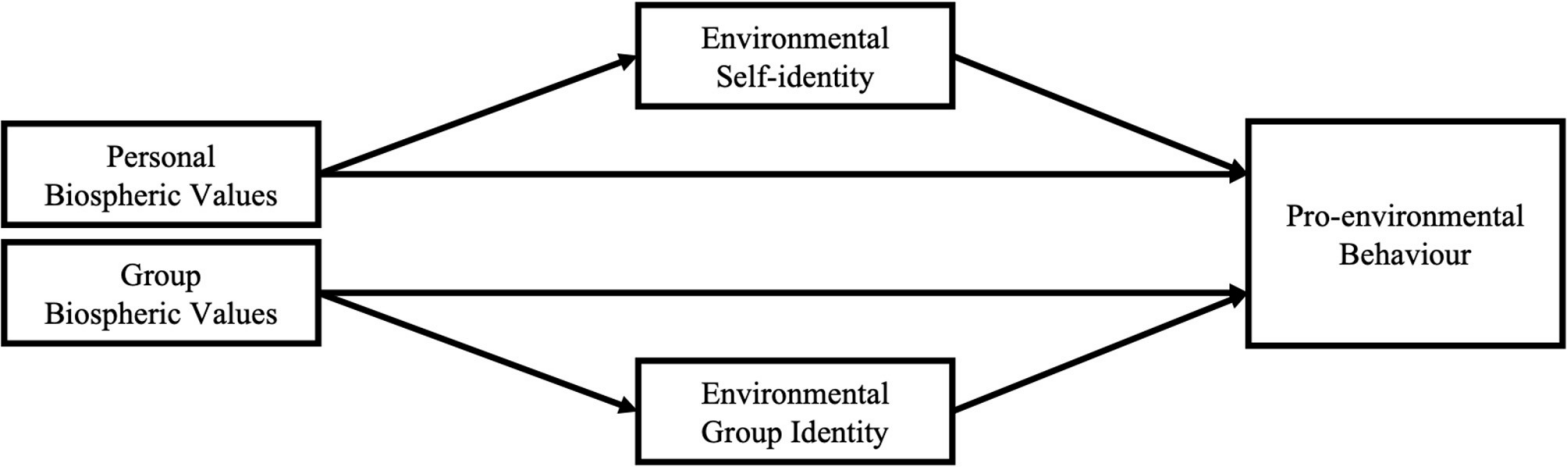
Personal and group pathways to predict pro-environmental behaviour.

In summary, the present study will test a personal and a novel group pathway to predict pro-environmental behaviour and explore their predictive power in participants from two countries: the Netherlands and China (see Figure 1). We hypothesise: at the personal level, personal biospheric values influence environmental self-identity (Hypothesis 1) and environmental self-identity, in turn, influences pro-environmental behaviour in both individual- and collective-oriented cultures (Hypothesis 2). At the group level, we hypothesise that group biospheric values influence environmental group identity (Hypothesis 3), which, in turn, will influence pro-environmental behaviour in both individual- and collective-oriented cultures (Hypothesis 4). In addition, we explore if the personal pathway would be more strongly related to pro-environmental behaviour in individualistic than in collectivistic cultures, whereas the group pathway might be more strongly related to pro-environmental behaviour in collectivistic than in individualistic cultures.

## Materials and Methods

### Participants and Procedure

A link for an online questionnaire in Dutch was sent out to the 1st-year psychology students in the Netherlands, and a Chinese questionnaire in hardcopy was sent to 1st-year business school students in China. For Chinese participants, we translated the relevant scales from English to Chinese (for values and identity). A detailed description of the translation process, which involved back translation, is provided in the Supplementary Materials. For Dutch participants, we used previously validated value and identity scales (Van der Werff et al., 2013a; Bouman et al., 2018). Questions for Chinese students were designed for this study only, but questions for Dutch students were part of a larger investigation; we only report data relevant for the present study. Participants from the Netherlands received course credits for their participation, while in China, no compensation was offered.

In both countries, participants were presented with the study introduction and started the online or hardcopy questionnaire after giving their informed consent. Questions on personal, perceived group values, environmental self-identity and group identity were presented, followed by the measures of their daily pro-environmental behaviour. Then, they were asked to indicate their product preference in a choice scenario; however, this measure was not used in the current study^2^. Results of how both pathways worked in environmental purchasing preference were similar as for pro-environmental behaviour.

In total, 169 students participated in the Netherlands, of whom 161 filled out all relevant questions for this study; 80% of the participants were female, and age ranged from 17 to 52 years old (*M* = 19.44, *SD* = 3.03). In China, 192 students participated in the study, of whom 168 filled out all relevant questions for this study; 71% were female. Participants' age ranged from 18 to 36 years old (*M* = 20.45, *SD* =2.44).

### Measures

#### Personal Biospheric Values

Participants were instructed to rate the importance of 16 items reflecting altruistic, egoistic, hedonic and biospheric values as “a guiding principle in their lives” on a scale from −1 = *opposed to my values*, 0 = *not important*, to 7 = *of supreme importance* based on a standard procedure (Schwartz, 1992). In the current study, we only used biospheric values, which were measured with four items: “respecting the earth,” “unity with nature,” “preventing pollution,” and “protecting the environment” (De Groot and Steg, 2007a; Steg et al., 2014). A multiple group method (MGM) confirmatory factor analysis was used to examine whether biospheric values could be distinguished empirically from the other values (De Groot and Steg, 2007b; Stuive et al., 2009). Results confirmed the validity of the value scales in the Netherlands and China (see Supplementary Materials for details). The reliability of the personal biospheric values scale was good in both the Netherlands (α = *0.9*0, *M* = 3.97, *SD* = 1.51) and China (α = *0.8*6, *M* = 4.79, *SD* = 1.31).

#### Group Biospheric Values

We measured group values with the same scale as personal values, but asking to rate the importance of the different values as a “guiding principle in your fellow students' lives,” i.e., concerning their fellow psychology (the Netherlands) or business school (China) students (e.g., Bouman et al., 2020a). Participants answered on the same scale from −1 = *opposed to my fellow students' values*, 0 = *not important*, to 7 = *of supreme importance*. The internal consistency of the group biospheric values scale was good (in the Netherlands: α = 0.86, *M* = 3.31, *SD* = 1.35; in China: α = 0.89, *M* = 4.36, *SD* = 1.38).

#### Environmental Self-Identity

Environmental self-identity was measured with a three-item scale (Van der Werff et al., 2013b): “I am the type of person who acts environmentally friendly,” “Acting environmentally friendly is an important part of who I am” and “I see myself as an environmentally friendly person.” Participants answered on a scale from 1 = *strongly disagree* to 7 = *strongly agree*. The internal consistency of the environmental self-identity scale was excellent (in the Netherlands: α = *0.9*3*, M* = 3.71, *SD* = 1.30; in China: α = *0.9*0, *M* = 4.96, *SD* = 1.02).

#### Environmental Group Identity

To measure the environmental group identity, we used a similar scale to the one measuring the environmental self-identity, but referring to the peers in their group. The three items were: “My Fellow psychology/business school students act environmentally friendly,” “Acting environmentally friendly is an important part of who my fellow psychology/business school students are” and “I see my fellow psychology/business school students as environmentally friendly.” Participants rated on a seven-point scale to what extent they agree with the items from 1 = *strongly disagree* to 7 = *strongly agree*. The internal consistency of the environmental group identity was excellent (in the Netherlands: α = *0.9*0, *M* = 3.16, *SD* = 1.10; in China: α = 94, *M* = 4.71, *SD* = 1.07).

#### Pro-environmental Behaviour

Pilot studies were conducted to establish common pro-environmental behaviour for people from both countries. Based on the commonly utilised scales in European countries (Barr, 2003; Cornelissen et al., 2008; Van der Werff et al., 2014a), we selected 13 items that are common to Chinese people as well. Participants from both countries were instructed to rate on a scale from 1 = *not at all* to 7 = *always* how frequently they engaged in each of them (see scales in Supplementary Materials). The internal consistency was good (in the Netherlands: α = *0.7*9, *M* = 4.75, *SD* =0.82; in China: α = *0.7*7, *M* = 5.06, *SD* =0.76).

## Results

### Correlations Between Biospheric Values, Environmental Identity, and Pro-environmental Behaviour

We first tested correlations between all relevant variables. In line with our predictions, most of the variables were positively related (see Table 1). Notably, personal and group biospheric values were strongly related, and so were environmental self- and group identities. Overall, correlations in the Netherlands and China were very similar. However, in the Netherlands, perceived group biospheric values were not significantly related to pro-environmental behaviour, while in China, they were. In addition, the relationship between personal biospheric values and environmental self-identity was significantly stronger in the Netherlands (*r* = 0.65, 95% CIs [0.55, 0.73]) than that in China (*r* = 0.37, 95% CIs [0.23, 0.49]).

**Table 1:** Bivariate correlations between personal and group biospheric values, environmental self- and group identities, and pro-environmental behaviour among Dutch students (highlighted in grey) and Chinese students in the main study.

	**1**	**2**	**3**	**4**	**5**
Personal biospheric values	-	0.67^**^	0.37^**^	0.22^**^	0.44^**^
Group biospheric values	0.62^**^	-	0.40^**^	0.50^**^	0.38^**^
Environmental self-identity	0.65^**^	0.33^**^	-	0.59^**^	0.56^**^
Environmental group identity	0.31^**^	0.35^**^	0.42^**^	-	0.33^**^
Pro-environmental behaviour	0.38^**^	0.11	0.53^**^	0.24^**^	-

** *p < 0.01*,

* *p < 0.05*.

### Model 1: Personal and Group Pathways in Predicting Pro-environmental Behaviour

To test if personal and group biospheric values affect pro-environmental behaviour via environmental self- or group identity, respectively, we applied bootstrap analysis with the PROCESS macro on SPSS 22.0 (Zhao et al., 2010; Hayes, 2013, 2016). We tested our model in two steps: in Model 1, we ran the analysis for the personal and group pathways separately (i.e., personal biospheric values and environmental self-identity, or group biospheric values and environmental group identity), in order to test each model's ability to predict pro-environmental behaviour. In Model 2, we tested both pathways together to examine how much variance the personal or group pathway uniquely explained when other variables from the other pathway were controlled for.

**Figure 2:**
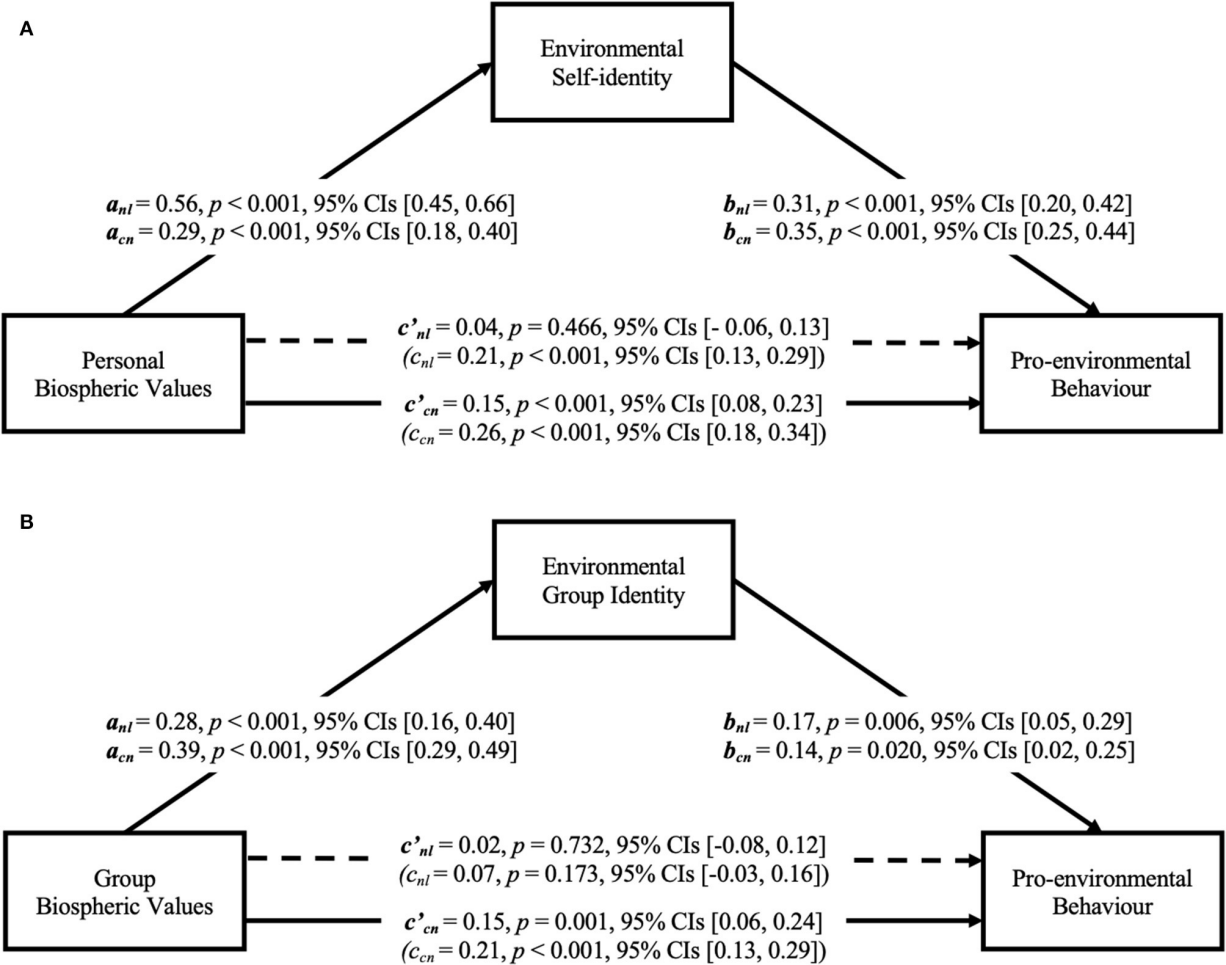
Indirect effects of biospheric values on pro-environmental behaviour via environmental identity at the personal **(A)** and group level **(B)** in Dutch and Chinese participants, bootstrap analysis. nl, Dutch participants; cn, Chinese participants; c, regression coefficients of personal biospheric values **(A)** or group biospheric values **(B)** on pro-environmental behaviour; c′, regression coefficients of personal biospheric values on pro-environmental behaviour with environmental self-identity involved **(A)**, or group biospheric values on pro-environmental behaviour with environmental group identity involved **(B)**.

As displayed in Figure 2A, we found support for our hypothesised personal-level pathway (Hypothesis 1 and Hypothesis 2): in both countries, stronger personal biospheric values were associated with a stronger environmental self-identity, and a stronger environmental self-identity was in turn related to more frequent engagement of pro-environmental behaviour.

In the Netherlands, this personal pathway explained 28% of the variance in pro-environmental behaviour, *F*_(2,158)_ = 30.99, *p* < 0.001. As expected, personal biospheric values were significantly related to pro-environmental behaviour; however, the direct association between personal biospheric values and pro-environmental behaviour became non-significant when controlling for environmental self-identity. The indirect effect via environmental self-identity was significant (*a* ^*^ *b* = 0.17, 95% CIs [0.10, 0.26]), which suggests there was an indirect-only mediation (Zhao et al., 2010). In line with our hypotheses, stronger personal biospheric values were indicative of more pro-environmental behaviour, and this relationship could be fully explained by biospheric values' positive association with environmental self-identity.

In China, personal biospheric values and environmental self-identity explained 38% of the variance in pro-environmental behaviour, *F*_(2,165)_ = 50.09, *p* < 0.001. There was a significant indirect effect of personal biospheric values on pro-environmental behaviour through environmental self-identity (*a* ^*^ *b* = 0.10, 95% CIs [0.06, 0.16]) as well; however, the direct effect of biospheric values on pro-environmental behaviour remained significant, which indicates a complementary mediation (Zhao et al., 2010). It provided evidence for our hypotheses: personal biospheric values could predict pro-environmental behaviour, and this seems to partially occur via strengthening environmental self-identity. However, complementary mediation suggests that there are likely to be other mediators as well.

In Figure 2B, we also found support for the novel group pathway (Hypothesis 3 and Hypothesis 4): stronger group biospheric values were associated with a stronger environmental group identity, and a stronger environmental group identity, in turn, was related to more pro-environmental behaviour in both countries.

In the Netherlands, group biospheric values and environmental group identity explained 6% of the variance in pro-environmental behaviour, *F*_(2,158)_ = 4.85, *p* = 0.009. The association between group biospheric values and pro-environmental behaviour was not significant; however, there was a significant indirect effect of group biospheric values on pro-environmental behaviour through environmental group identity (*a* ^*^ *b* = 0.05, 95% CIs [0.01, 0.11]). Therefore, it is an indirect-only mediation (Zhao et al., 2010): group biospheric values predicted pro-environmental behaviour fully via environmental group identity in Dutch samples.

In China, group biospheric values and environmental group identity explained 17% of the variance in pro-environmental behaviour, *F*_(2,165)_ = 16.79, *p* < 0.001. There was also a significant indirect effect of group biospheric values on pro-environmental behaviour through environmental group identity (*a* ^*^ *b* = 0.05, 95% CIs [0.01, 0.11]). The direct effect of group biospheric values on pro-environmental behaviour was significant, which suggests a complementary mediation again (Zhao et al., 2010). This suggests that there are likely to be other mediators that explain the relationship between group biospheric values and pro-environmental behaviour in Chinese samples as well.

### Model 2: Personal and Group Pathways in Predicting Pro-environmental Behaviour

**Figure 3:**
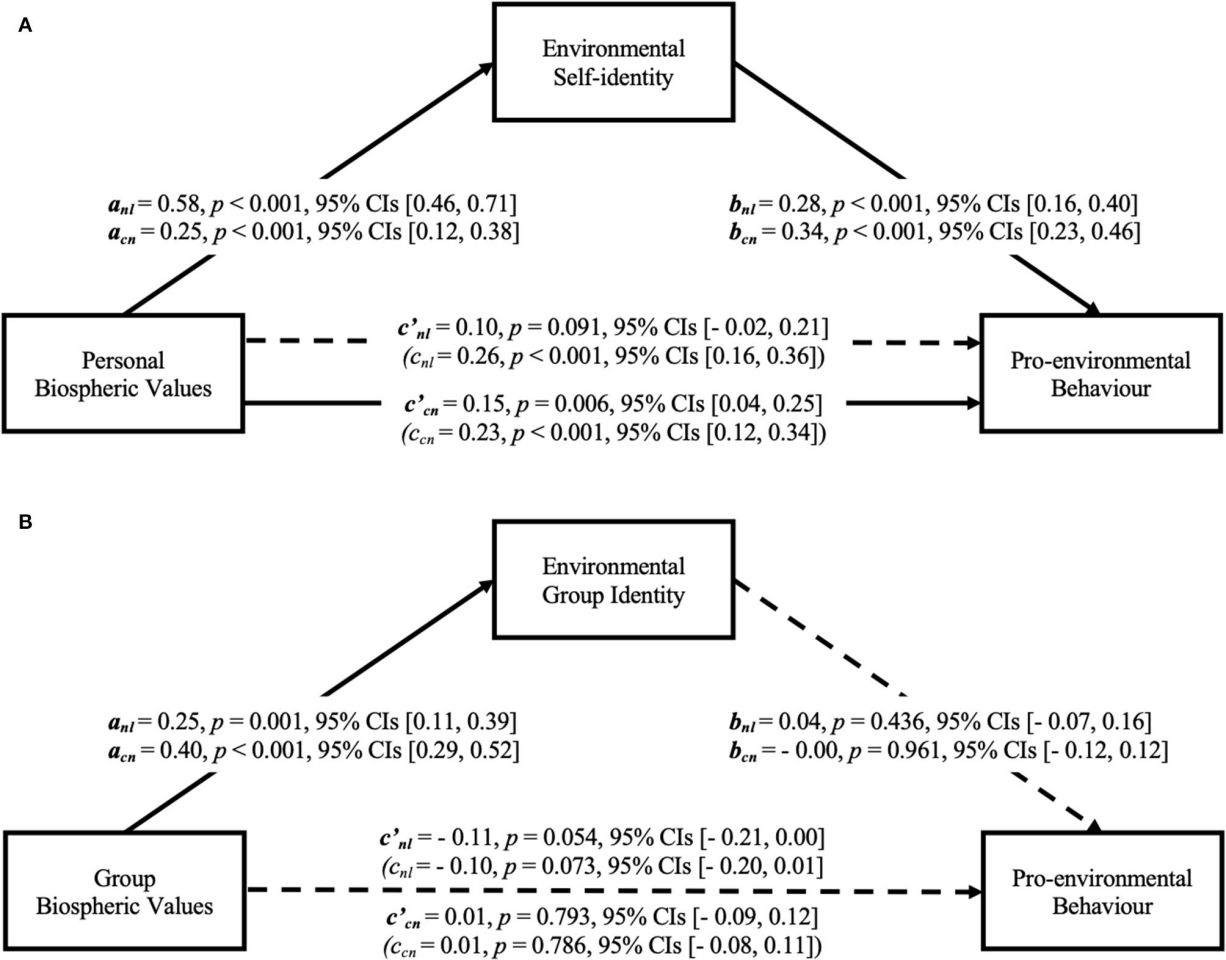
Indirect effects of biospheric values on pro-environmental behaviour via environmental identity at the personal level, controlling for group-level variables **(A)**, and at the group level, controlling for personal-level variables **(B)**, in Dutch and Chinese participants, bootstrap analysis. nl, Dutch participants; cn, Chinese participants; c, regression coefficients of personal biospheric values **(A)** or group biospheric values **(B)** on pro-environmental behaviour, controlling for variables from the other pathway; c′, regression coefficients of personal biospheric values on pro-environmental behaviour with environmental self-identity involved **(A)**, controlling for group-level variables; or group biospheric values on pro-environmental behaviour with environmental group identity involved **(B)**, controlling for personal-level variables.

The full model (Model 2) tested both pathways together by including variables from personal and group levels, in order to reveal unique contributions of each pathway (see Figure 3). Results showed that the personal pathway remained significant; however, the group pathway did not explain unique explained variance when controlling for the personal level variables. In the Netherlands, Model 2 explained 30% of the variance in pro-environmental behaviour, *F*_(4,156)_ = 16.64, *p* < 0.001; and in China, the model explained 38% of the variance in pro-environmental behaviour, *F*_(4,163)_ = 24.77, *p* < 0.001.

As shown in Figure 3A, we found support for the personal pathway when controlling for the group-level factors (Hypothesis 1 and Hypothesis 2): personal biospheric values were associated with environmental self-identity, and environmental self-identity was in turn positively related to pro-environmental behaviour when we controlled for group biospheric values and environmental group identity. In the Netherlands, personal biospheric values were still indirectly related to pro-environmental behaviour via environmental self-identity (*a* ^*^ *b* = 0.16, 95% CIs [0.09, 0.26]), even when variables at the group level were controlled for. The direct effect was not significant, which suggests an indirect-only mediation (Zhao et al., 2010). In China, the indirect effect of personal biospheric values on pro-environmental behaviour through environmental self-identity was significant (*a* ^*^ *b* = 0.09, 95% CIs [0.04, 0.16]) when variables at the group level were controlled for. The direct effect was still significant, which suggests a complementary mediation, and there may be other mediators (Zhao et al., 2010).

However, in Model 2, when personal biospheric values and environmental self-identity were controlled for, the group biospheric values were related to environmental group identity in samples from both countries, but not to pro-environmental behaviour. We did not find a significant relationship between environmental group identity and pro-environmental behaviour either. The indirect effect of group biospheric values on pro-environmental behaviour through environmental group identity was not significant in the Netherlands (*a* ^*^ *b* = 0.01, 95% CIs [−0.01, 0.05]) or in China (*a* ^*^ *b* = 0.00, 95% CIs [−0.06 to 0.05]) when controlling for the personal-level variables.

## Discussion

### Personal Pathway and Group Pathway Across Countries

This study aimed to test and compare an existing personal and a novel group pathway to explain pro-environmental behaviour in the Netherlands and China. Specifically, in the personal pathway, we tested if personal biospheric values influenced pro-environmental behaviour via one's environmental self-identity. In the group pathway, we proposed and tested whether group biospheric values influenced pro-environmental behaviour via one's environmental group identity. Overall, we found support for the personal and group pathways in participants from both countries. Stronger personal biospheric values were associated with a stronger environmental self-identity, and a stronger environmental self-identity, in turn, encouraged pro-environmental behaviour. Importantly, we also found support for the group pathway in participants from two countries. Stronger group biospheric values were associated with a stronger environmental group identity, and a stronger environmental group identity also encouraged pro-environmental behaviour. However, when we tested both pathways together, we found that the personal pathway uniquely explained variance in pro-environmental behaviour while the group pathway did not.

Our finding that the personal pathway explains pro-environmental behaviour in a specific sample in an individualistic country is in line with previous research (Gatersleben et al., 2012; Van der Werff et al., 2013a, 2014b) and extends it to a specific sample from a collectivistic country, namely, China. Interestingly, although in both samples we found support for the hypothesised indirect relationship between personal biospheric values and pro-environmental behaviour via environmental self-identity, we observed differences in the strength in which personal biospheric values were directly and indirectly associated with pro-environmental behaviour in participants from two countries. For Dutch students, biospheric values related to pro-environmental behaviour fully via environmental self-identity, to which biospheric values were relatively strongly associated. For Chinese students, the association between biospheric values and environmental self-identity was considerably weaker, and personal biospheric values were also found to directly (i.e., not via environmental self-identity) relate to pro-environmental behaviour here. These differences could be due to cultural differences. Western cultures foster individuals being different from others, while East Asian cultures tend to foster individuals as interdependent with others (Markus and Kitayama, 1991; Vignoles et al., 2016). Accordingly, for our Dutch participants, personal biospheric values could be interpreted as something that distinguishes oneself from others, and thus being more strongly connected with how individuals see themselves; while for Chinese participants, the personal biospheric values could be interpreted as something that aligns individuals with others, thus being less strongly connected with how they see themselves. Therefore, in western countries, personal values may influence pro-environmental behaviour fully through self-identity. In the East Asian context, there may be other reasons why endorsing biospheric values would motivate individuals to engage more in pro-environmental behaviour besides environmental self-identity, such as group factors (e.g., social influence, Schultz et al., 2008). For future research, it is worth investigating other potential factors that link personal biospheric values and pro-environmental behaviour in a more collectivistic culture.

In addition, we found support for the group pathway in our samples from both countries, and results indicated that it might be more predictive of pro-environmental behaviour among Chinese students than among Dutch students. Our study thus provides convergent evidence for the group and social factors influencing environmental behaviour (Schultz et al., 2008; Laidley, 2013; Masson and Fritsche, 2014). Together with recent studies discussing such group-based approaches (Jans et al., 2018; Bouman and Steg, 2019, 2020), our findings support the possible relations between group biospheric values and pro-environmental cross-culturally and provide insights in the process through which values motivate pro-environmental behaviour. Moreover, our studies imply that group values and group identity are worth investigating, particularly when conducting studies in a collectivistic culture.

Although we found support for our novel group pathway, it is also important to note that the group pathway was less strongly related to pro-environmental behaviour than the personal pathway. Moreover, when we inspected both pathways together, the personal pathway uniquely explained variance in pro-environmental behaviour while the group pathway did not. The observation that single group factors may be less predictive of personal behaviour than corresponding personal factors is in line with earlier research (e.g., Bamberg et al., 2007; Bouman et al., 2020b). Individuals can be influenced by many different groups, and the influence of group values and norms likely depends on many different factors (e.g., identification with the group, relevance of the group for the behaviour, Tajfel and Turner, 1979), which may explain why the groups we selected in our studies had a relatively weak effect on the participants' behaviour. In addition, most measured pro-environmental behaviours were personal and private, and thus measured at the same conceptual level as the personal pathway variables. Arguably, the influence of group factors may have been larger for more collective actions. Accordingly, future research could try to replicate our study with other groups and other behaviours, particularly to investigate whether effect sizes of the group pathway will be stronger when the group is more relevant and when the measured behaviours are more socially oriented.

Importantly, personal and group factors were also related to each other, which explains why effects of group factors may appear less important when controlling for personal-level factors. Interestingly, however, our result also suggests that perceived group values and identity may influence pro-environmental behaviour via the personal pathway, particularly in our Chinese sample. Specifically, the group biospheric values and environmental group identity may influence the environmental self-identity and thereby promote pro-environmental behaviour. This observation could be interpreted as being in line with earlier theorising, which suggests that self-identity development is influenced by others and in-groups (Cooley, 1902; Tajfel and Turner, 1979; Smith and Henry, 1996). Yet, future research is needed to test if the group pathway indeed influences environmental behaviour via the personal pathway.

In addition, to better understand how our novel group pathway may contribute to the existing literature, it is essential to elaborate on how group biospheric values and environmental group identity differ from more frequently studied group factors, such as descriptive and injunctive group norms. Specifically, whereas group values and group identity are respectively about what is important and defining for a group, group norms are about what behaviours are approved (i.e., injunctive norm) and commonly performed (i.e., descriptive norm) by a group (Cialdini et al., 1990). Although these constructs relate to each other—pro-environmental group norms will likely be stronger when there are stronger group biospheric values and when there is a stronger environmental group identity (and vice versa)—this does not always have to be the case. For example, not all commonly performed behaviours (i.e., descriptive norms) are defining for a group's identity. Importantly, we rather see group values and a corresponding group identity to underlie group norms, explaining why group members (dis)approve and perform certain behaviours. More research is needed on this, in particular on how these constructs relate to each other and how they could be teased apart. The latter may also be important considering some overlap in specific items used to measure identity and norms, which appears undesirable according to the abovementioned reasoning.

Generally, we found that the results for personal and group pathways were rather similar in the student samples from the Netherlands and China, which also has a few implications. Importantly, it suggests that both of our pathways have good generalisability, as the results were stable across different groups of participants (i.e., psychology vs. business students), in different countries (i.e., Netherlands vs. China). It is however important to note that more studies are needed with more national representative samples to draw conclusions about specific countries and cultures, and whether these differ from each other. Our specific sample of Chinese business students may be relatively individualistic compared to other Chinese citizens, whereas our Dutch psychology students may have been more socially oriented than other Dutch citizens. Hence, whereas our data provide first evidence that the personal and group pathway exist across populations, more research is needed to investigate potential cultural and country differences.

The current study also had some limitations. First, it was a correlational study; therefore, no causal conclusions can be drawn. Future research is needed to test the potential causal pathways via experimental design where one or more environmental identities are manipulated to see if it can indeed improve environmental relevant behaviour across countries. Second, we used self-reported measures for pro-environmental behaviour in this study. A future study could also investigate actual behaviour, such as measuring food waste recycling behaviour.

### Implications for Practitioners

Based on our findings, it might be worthwhile to aim to strengthen personal and group biospheric values, as well as environmental self- and group identity to encourage pro-environmental behaviour in individualistic and collectivistic countries. Specifically, our study supports the relationship between environmental self-identity and pro-environmental behaviour across cultures. Previous studies suggest that reinforcing environmental self-identity helps to promote environmental relevant behaviour (Van der Werff et al., 2014a). To promote environmental behaviour, practitioners may try to make the environmental self-identity salient for the targeted participants by making people's past pro-environmental actions salient (Cornelissen et al., 2008; Van der Werff and Steg, 2016).

The existence of a group-level pathway also suggests that interventions targeting the perceived group values and identity could be promising to promote pro-environmental behaviour. Policymakers could deliver a message emphasising that group members do care about the environment and which characterises the group as “pro-environmental.” It is noteworthy that this is different from a message based on social norms, which would communicate instead that the group finds it important that members act pro-environmental (injunctive norms) or merely that the group acts pro-environmental (descriptive norms). For instance, a neighbourhood energy-saving project may try to convey messages as “we do care about conserving natural resources” and “conservative energy use is an important part of what our community is.”

More importantly, group-level predictors might be easier to adjust than personal-level predictors (see Bouman and Steg, 2019, 2020). Whereas, people might feel they know best what they themselves find important, their perceptions of the group might be more open to being influenced by information they receive from others, which suggests the group approach's potential.

## Conclusion

In conclusion, our results indicate support for the well-established personal pathway and, to a lesser extent, for a newly proposed group pathway. Specifically, we replicated earlier findings that personal biospheric values can, via environmental self-identity, predict pro-environmental behaviour and extended these findings to participants from a collectivistic culture. Moreover, we found support for our hypothesised group pathway in participants from both countries, in which biospheric group values relate to pro-environmental behaviour via environmental group identity, although its effects were considerably weaker than for the personal pathway.

## Data Availability Statement

The raw data supporting the conclusions of this article will be made available by the authors for research purposes, without undue reservation.

## Ethics Statement

The studies involving human participants were reviewed and approved by Ethical Committee of Psychology, University of Groningen. The patients/participants provided their written informed consent to participate in this study.

## Author Contributions

XW, EV, TB, MH, and LS conceptualised the research model. XW, EV, and TB designed the study and collected and analysed the data. XW, EV, TB, and MH drafted the manuscript. EV, TB, MH, and LS engaged in critical revisions of the manuscript. All authors contributed to the article and approved the submitted version.

## Conflict of Interest

The authors declare that the research was conducted in the absence of any commercial or financial relationships that could be construed as a potential conflict of interest.

## Footnotes

^1^Please note that this does not mean we expect the group pathway to be stronger than the personal pathway in China, which we believe is unlikely considering earlier research (e.g., Bamberg et al., 2007; Bouman et al., 2020b; see also Discussion section).

^2^The purchasing preference scenario was our measure for pro-environmental behaviour as well. It was measured directly after participants reporting their daily pro-environmental behaviour, which might have changed participants' environmental identity or other antecedents influencing the behaviour (Van der Werff et al., 2014a). Besides, we found similar but relatively weaker effects of both pathways on the purchasing preference. We did not report the results in the main paper. Details can be found in Supplementary Data.
