# Supplementary material for: I Am vs. We Are: How Biospheric Values and Environmental Identity of Individuals and Groups Can Influence Pro-environmental Behaviour
Source: Front Psychol. 2021 Feb 18;12:618956. doi: 10.3389/fpsyg.2021.618956 (PMC7930912; doi:10.3389/fpsyg.2021.618956)
Supplement: Supplementary file 1 [file Data_Sheet_1.docx]

Supplementary Material

Contents

[1 Biospheric Value Scales Translation & Validity Test in China 1](#_Toc57991108)

[2 Measures of Key Variables 3](#_Toc57991109)

[2.1 Value Scales (English and Chinese) 3](#_Toc57991110)

[2.2 Environmental Identity 8](#_Toc57991111)

[2.3 Pro-Environmental Behaviour 9](#_Toc57991112)

[2.4 Environmental Purchasing Preference 10](#_Toc57991113)

[3 Mediation Effect Analysis Results of Personal and Group Pathway for Pro-environmental Purchasing Preference 11](#_Toc57991114)

[4 References 14](#_Toc57991115)

# Biospheric Value Scales Translation & Validity Test in China

We translated the value scales from English to Chinese. To inspect whether the translated items were understandable and meaningful in the local context, we conducted a pilot study in Shanghai, China, in March 2014. Each participant received a bag of salt as a small gift. The questionnaire was presented to 22 residents in a relaxing common space with investigators standing by. When residents had problems understanding each value item, we would ask which words/phrases were confusing and then try to let participants come to their conclusions instead of offering a direct explanation. We adapted the value items accordingly. Later, a back-translation was checked by two translators. Before conducting the main study, we tested the value scales among students (*N* = 66). The results of this test can be found in the supplementary materials. The results show the four values can be distinguished in the Chinese sample.

We used the value scale to measure all four values orientations in the main study as well, namely egoistic values, altruistic values, hedonic values, and biospheric values. The multiple group method (MGM) (De Groot & Steg, 2007; Stuive et al., 2009) was used to examine whether the four values orientations were distinguished into different groups, respectively. Results showed that the items indeed clustered into four the value orientations as expected. The analysis confirmed the validity of the value scales in China, suggesting the four values can be distinguished in China.

Specifically, the biospheric value items correlated strongest with the corresponding value orientation. The Cronbach’s alpha was good both at the personal level (*α* = .86, *M* = 4.93, *SD* =1.32), and at the group level (*α* = .89, *M* = 4.36, *SD* = 1.38) (see Table 1).

**Table 1**. Corrected correlations between value items and value clusters via multiple group method (MGM) among Chinese students (*N* = 168)

|  | **Value Cluster** | | | |
| --- | --- | --- | --- | --- |
| **Personal Biospheric Values** | **Hedonic** | **Egoistic** | **Altruistic** | **Biospheric** |
| 1. Respecting the earth | .15 | .11 | .41 | **.64** |
| 1. Unity with nature | .30 | .29 | .54 | **.70** |
| 1. Protecting the environment | .39 | .26 | .60 | **.80** |
| 1. Preventing pollution | .37 | .28 | .56 | **.68** |
| **Group Biospheric Values** |  | | | |
| 1. Respecting the earth | .40 | .22 | .691 | **.692** |
| 1. Unity with nature | .57 | .25 | .67 | **.76** |
| 1. Protecting the environment | .54 | .28 | .70 | **.76** |
| 1. Preventing pollution | .61 | .30 | .75 | **.78** |

*Note.* Correlation coefficients are corrected for self-correlations. Highest correlations for each value item are printed in bold.

# Measures of Key Variables

## Value Scales (English and Chinese)

(The differences of measuring personal values and group values are in the instructions only.)

### English Value Scales

Instructions **for INDIVIDUALS:**

Below you will find 16 values, behind each value there is a short explanation concerning the meaning of the value. Could you please rate how important each value is for you **AS A GUIDING PRINCIPLE IN YOUR LIFE**?

The rating scale is as follows:

0 mean the value is *not important* at all; it is not relevant as a guiding principle in your life

3 means the value is *important*

6 means the values is *very important*

-1 mean the value is *opposed* to the principles that guide you

7 means the value is of *supreme importance* as a guiding principle in your life;

Ordinarily there are no more than two such values

Your scores can vary from -1 up to 7. The higher the number (-1, 0, 1, 2, 3, 4, 5, 6, 7), the more important the value is as a guiding principle in YOUR life. Try to distinguish as much as possible between your ratings of the values by using different numbers.

Instructions **for GROUPS:**

On the previous page you indicated how important various values are in your life. How important do you think those values are, on average, for your fellow students? In other words, please rate how important each value is, on average, as a **GUIDING PRINCIPLE IN YOUR FELLOW STUDENTS’ LIVES**.

The rating scale is as follows:

0 means the value is *not important* at all as a guiding principle in your fellow students’ lives.

3 means the value is *important* as a guiding principle in your fellow students’ lives.

6 means the value is *very important* as a guiding principle in your fellow students’ lives.

-1 means the value is *opposed to* the principles of your fellow students.

7 means the value is *of supreme importance* as a guiding principle in your fellow students’ lives.

Ordinarily there are no more than two such values

Your scores can vary from -1 up to 7. The higher the number (-1, 0, 1, 2, 3, 4, 5, 6, 7), the more important the value is as a guiding principle in fellow students’ lives. Try to distinguish as much as possible between your ratings of the values by using different numbers.

|  | **Opposed to my (our) values**  **-1** | **0** | **1** | **2** | **Important**  **3** | **4** | **5** | **6** | **Of supreme importance**  **7** |
| --- | --- | --- | --- | --- | --- | --- | --- | --- | --- |
| 1. EQUALITY: equal opportunities for all | □ | □ | □ | □ | □ | □ | □ | □ | □ |
| 1. RESPECTING THE EARTH: harmony with other species | □ | □ | □ | □ | □ | □ | □ | □ | □ |
| 1. SOCIAL POWER: control over others, dominance | □ | □ | □ | □ | □ | □ | □ | □ | □ |
| 1. PLEASURE: joy, gratification of desires | □ | □ | □ | □ | □ | □ | □ | □ | □ |
| 1. UNITY WITH NATURE: fitting into nature | □ | □ | □ | □ | □ | □ | □ | □ | □ |
| 1. A WORLD AT PEACE: free of war and conflict | □ | □ | □ | □ | □ | □ | □ | □ | □ |
| 1. WEALTH: material possessions, money | □ | □ | □ | □ | □ | □ | □ | □ | □ |
| 1. AUTHORITY: the right to lead or command | □ | □ | □ | □ | □ | □ | □ | □ | □ |
| 1. SOCIAL JUSTICE: correcting injustice, care for the weak | □ | □ | □ | □ | □ | □ | □ | □ | □ |
| 1. ENJOYING LIFE: enjoying food, sex, leisure, etc. | □ | □ | □ | □ | □ | □ | □ | □ | □ |
| 1. PROTECTING THE ENVIRONMENT: preserving nature | □ | □ | □ | □ | □ | □ | □ | □ | □ |
| 1. INFLUENTIAL: having an impact on people and events | □ | □ | □ | □ | □ | □ | □ | □ | □ |
| 1. HELPFUL: working for the welfare of others | □ | □ | □ | □ | □ | □ | □ | □ | □ |
| 1. PREVENTING POLLUTION: protecting natural resources | □ | □ | □ | □ | □ | □ | □ | □ | □ |
| 1. SELF-INDULGENT: doing pleasant things | □ | □ | □ | □ | □ | □ | □ | □ | □ |
| 1. AMBITIOUS: hard working, aspiring | □ | □ | □ | □ | □ | □ | □ | □ | □ |

##

### Chinese Value Scales

Instruction **for INDIVIDUALS:**

以下为16项价值指标及描述，每一个指标后都有对这个指标内容的简短解释。您能否根据每个指标作为您生活引导原则的重要性进行打分？您给出的分数可从-1至7(-1,0,1,2,3,4,5,6,7)。分数越高则代表该价值观作为您生活中指导原则的重要性越高。请尽可能利用不同的分数区分不同指标对您的重要程度。

**0** 完全不重要；与您的价值观念不相关

**3** 重要

**6** 十分重要

**-1** 所列项与您的人生价值背离

**7** 所列项是您人生中极为重要的价值指导；通常不超过两项

Instructions **for GROUPS:**

上题中，您标记了每项指标作为人生指导原则对您个人的重要性，本题中，您需要指出这些价值观在**您的同学（同一院系）**中通常有多重要？请为每一个价值观打分，显示它们作为**您同学们的生活指导原则**中有多重要。您给出的分数可从-1至7(-1,0,1,2,3,4,5,6,7)。分数越高则代表价值指标作为您学院同学生活中指导原则的重要性越高。请尽可能利用不同的分数区分不同指标对您的重要程度。

**0** 完全不重要

**3** 重要

**6** 十分重要

**-1** 所列项与您所在学院同学们的人生价值背离

**7** 所列项是您所在同学们人生中极为重要的价值指导；通常不超过两项

|  | **背离我（其）的价值观念-1** | **0** | **1** | **2** | **重要**  **3** | **4** | **5** | **6** | **极为重要 7** |
| --- | --- | --- | --- | --- | --- | --- | --- | --- | --- |
| **1. 平等**：人人机会均等。 | □ | □ | □ | □ | □ | □ | □ | □ | □ |
| **2. 爱护地球：**尊重其他的物种（动物、植物等），与其他物种和谐相处。 | □ | □ | □ | □ | □ | □ | □ | □ | □ |
| **3. 社会权力：**管理和支配他人，主导、优势地位。 | □ | □ | □ | □ | □ | □ | □ | □ | □ |
| **4. 快乐：**满足自己的心愿。 | □ | □ | □ | □ | □ | □ | □ | □ | □ |
| **5. 与自然的和谐统一：**适应并融入自然。 | □ | □ | □ | □ | □ | □ | □ | □ | □ |
| **6. 世界和平：**没有战争和冲突的世界。 | □ | □ | □ | □ | □ | □ | □ | □ | □ |
| **7. 财富：**物质财产，金钱。 | □ | □ | □ | □ | □ | □ | □ | □ | □ |
| **8. 权威：**拥有领导和指挥他人的权利。 | □ | □ | □ | □ | □ | □ | □ | □ | □ |
| **9. 社会公平正义：**伸张正义，关心爱护弱者。 | □ | □ | □ | □ | □ | □ | □ | □ | □ |
| **10. 享受生活：**享受美食，休闲娱乐等。 | □ | □ | □ | □ | □ | □ | □ | □ | □ |
| **11. 保护环境：**维护良好的自然生态环境。 | □ | □ | □ | □ | □ | □ | □ | □ | □ |
| **12. 影响力：**有影响他人、事件的能力。 | □ | □ | □ | □ | □ | □ | □ | □ | □ |
| **13. 乐于助人：**帮助他人得到幸福。 | □ | □ | □ | □ | □ | □ | □ | □ | □ |
| **14. 防止污染：**保护与合理利用自然资源。 | □ | □ | □ | □ | □ | □ | □ | □ | □ |
| **15. 享乐：**做令自己愉快和享受的事。 | □ | □ | □ | □ | □ | □ | □ | □ | □ |
| **16. 雄心壮志：**勤奋努力，有志向、有抱负。 | □ | □ | □ | □ | □ | □ | □ | □ | □ |

## Environmental Identity

### Please indicate to what extent you agree with the following statements (Environmental self-identity):

|  | **Strongly disagree 1** | **2** | **3** | **4** | **5** | **6** | **Strongly agree 7** |
| --- | --- | --- | --- | --- | --- | --- | --- |
| I am the type of person who acts environmentally- friendly | ○ | ○ | ○ | ○ | ○ | ○ | ○ |
| Acting environmentally- friendly is an important part of who I am | ○ | ○ | ○ | ○ | ○ | ○ | ○ |
| I see myself as an environmentally- friendly person | ○ | ○ | ○ | ○ | ○ | ○ | ○ |

### Now please think about your fellow students. Please indicate to what extent you agree with the following statements (environmental group identity):

|  | **Strongly disagree 1** | **2** | **3** | **4** | **5** | **6** | **Strongly agree 7** |
| --- | --- | --- | --- | --- | --- | --- | --- |
| My Fellow business/psychology school students act environmentally- friendly | ○ | ○ | ○ | ○ | ○ | ○ | ○ |
| Acting environmentally- friendly is an important part of who my fellow business/psychology school students are | ○ | ○ | ○ | ○ | ○ | ○ | ○ |
| I see my fellow business/psychology school students as environmentally- friendly | ○ | ○ | ○ | ○ | ○ | ○ | ○ |

## Pro-Environmental Behaviour

Below you will find several different types behaviours. Please indicate to what extent you agree with the questions on behaviours in your daily life? From 1 = totally disagree to 7= totally agree.

|  | Totally disagree **1** | **2** | **3** | **4** | **5** | **6** | Totally agree **7** |
| --- | --- | --- | --- | --- | --- | --- | --- |
| 1. I separate paper from my waste | ○ | ○ | ○ | ○ | ○ | ○ | ○ |
| 1. I recycle glass | ○ | ○ | ○ | ○ | ○ | ○ | ○ |
| 1. I use paper economically | ○ | ○ | ○ | ○ | ○ | ○ | ○ |
| 1. I only use the washing machine when there is full load of laundry | ○ | ○ | ○ | ○ | ○ | ○ | ○ |
| 1. I switch off the *heater/air conditioner* when I leave my house | ○ | ○ | ○ | ○ | ○ | ○ | ○ |
| 1. I turn off the tap when washing dishes | ○ | ○ | ○ | ○ | ○ | ○ | ○ |
| 1. I use energy saving electronic household appliances | ○ | ○ | ○ | ○ | ○ | ○ | ○ |
| 1. I shut down the lights when leaving the room | ○ | ○ | ○ | ○ | ○ | ○ | ○ |
| 1. I prefer products with less packaging | ○ | ○ | ○ | ○ | ○ | ○ | ○ |
| 1. I use my own bag when shopping | ○ | ○ | ○ | ○ | ○ | ○ | ○ |
| 1. I throw litter on the street (score reversed) | ○ | ○ | ○ | ○ | ○ | ○ | ○ |
| 1. I pick up the litter when seeing it on the street | ○ | ○ | ○ | ○ | ○ | ○ | ○ |
| 1. I encourage others to recycle | ○ | ○ | ○ | ○ | ○ | ○ | ○ |

## Environmental Purchasing Preference

Imagine that you want to buy the products described below. For every product you can choose between 2 options. The first option is cheap, but not sustainably produced. The second option is sustainably produced, but more expensive. Please indicate for each product which option you would choose.

1. A designer pair of jeans of *100 euros / 450 yuan*, which is produced unsustainably.

A designer pair of jeans of *110 euros / 495 yuan*, which is produced sustainably.

1. A liter of milk of *65 cents / 8.90 yuan*, which is produced unsustainably.

A liter of milk of *72 cents / 9.80 yuan*, which is produced sustainably.

1. A new laptop of *900 euros / 6500 yuan*, which is produced unsustainably.

A new laptop of *990 euros / 7150 yuan*, which is produced sustainably.,

1. A pen of *1 euro / 1.50 yuan*, which is produced unsustainably.

A pen of *1.1 euros / 1.65 yuan*, which is produced sustainably.

1. A writing pad of *3 euros / 7.00 yuan*, which is produced unsustainably.

A writing pad of *3.3 euros / 7.70 yuan,* which is produced sustainably.

1. A bicycle of *400 euros / 500 yuan*, which is produced unsustainably.

A bicycle of *440 euros / 550 yuan*, which is produced sustainably.

1. A pair of socks of *3 euros / 8.00 yuan*, which is produced unsustainably.

A pair of socks of *3.3 euros / 8.80 yuan*, which is produced sustainably.

1. The newest model mobile phone of *500 euros / 3700 yuan*, which is produced unsustainably.

The newest model mobile phone of *550 euros / 4070 yuan*, which is produced sustainably.

# Mediation Effect Analysis Results of Personal and Group Pathway for Pro-environmental Purchasing Preference

*Environmental purchasing preference.* Participants were instructed in a product choice scenario to choose between eight paired products. In the scenario, of each paired product one product was always less expensive but unsustainably produced and the other was 10% more expensive but sustainably produced (Van der Werff et al., 2014). We counted the number of sustainably produced products chosen. In the Netherlands, participants chose on average 3.86 (*SD* = 2.17), and in China 5.53 (*SD* = 2.26) out of 8 sustainable products. In Table 2, we presented the mediation analysis of personal and group pathways for two Models as in the main study on purchasing preference.

**Table 2.** Indirect effects of biospheric values in predicting environmental purchasing preference via the environmental identity at the individual and group levels in the Netherlands and in China, bootstrap analysis.

|  |  | Model 1 | | | | Model 2 | | | |
| --- | --- | --- | --- | --- | --- | --- | --- | --- | --- |
|  |  | Individual Pathway | | Group Pathway | | Individual Pathway | | Group Pathway | |
|  |  | The Netherlands | China | The Netherlands | China | The Netherlands | China | The Netherlands | China |
| *a* | **Values – Identity** | **.56*****  (10.72) | **.29*****  (5.19) | **.28*****  (4.67) | **.39*****  (7.52) | **.58*****  (9.28) | **.25*****  (3.89) | **.25*****  (3.38) | **.40*****  (6.72) |
|  | *p* | < .001 | < .001 | < .001 | < .001 | < .001 | < .001 | < .001 | < .001 |
|  | 95% CIs | [.45, .66] | [.18, .40] | [.16, .40] | [.29, .49] | [.46, .71] | [.12, .38] | [.10, .39] | [.29, .52] |
| *b* | **Identity – Purchasing** | **.67*****  (4.26) | **.39***  (2.19) | **.38***  (2.34) | .20  (1.08) | **.61*****  (3.63) | .36  (1.64) | .12  (.71) | .09  (.39) |
|  | *p* | < .001 | .030 | .020 | .282 | < .001 | .103 | .477 | .695 |
|  | 95% CIs | [.36, .98] | [.04, .74] | [.06, .71] | [-.17, .57] | [.28, .95] | [-.07, .79] | [-.20, .43] | [-.35, .53] |
| *c* | **Values – Purchasing, Total Effect** | **.43*****  (3.92) | **.36****  (2.75) | .16  (1.29) | .25  (1.96) | **.51*****  (3.72) | **.39***  (2.17) | -.15  (-.98) | -.05  (-.30) |
|  | *p* | < .001 | .007 | .201 | .052 | < .001 | .031 | .330 | .765 |
|  | 95% CIs | [.21, .64] | [.10, .62] | [-.09, .41] | [-.00, .50] | [.24, .79] | [.04, .75] | [-.44, .15] | [-.39, .29] |
| *c’* | **Values – Purchasing, Direct Effect** | .05  (.38) | .25  (1.76) | .05  (.41) | .17  (1.15) | .16  (.94) | .30  (1.61) | -.17  (-1.13) | -.09  (-.45) |
|  | *p* | .701 | .080 | .684 | .252 | .349 | .109 | .262 | .655 |
|  | 95% CIs | [-.22, .32] | [-.03, .52] | [-.21, .32] | [-.12, .46] | [-.17, .48] | [-.07, .67] | [-.48, .13] | [-.47, .30] |
| *a * b* | **Values – Purchasing, Indirect Effect** | **.37** | **.11** | **.11** | .08 | **.36** | .09 | .03 | .04 |
|  | 95% CIs | [.19, .59] | [.02, .26] | [.02, .25] | [-.05, .22] | [.16, .60] | [-.00, .23] | [-.04, .13] | [-.11, .20] |
| *R^2^* |  | 18% | 7% | 4% | 3% | 19% | 7% | 19% | 7% |
| Mediation type |  | Indirect Only | Indirect Only | Indirect Only | Non-mediation | Indirect Only | Non-mediation | Non-mediation | Non-mediation |

Notes:

1. t-values are presented in parentheses.
2. a refers to the regression coefficients of biospheric values on the environmental identity at the personal or group level.
3. b refers to the regression coefficients of the environmental identity on environmental purchasing preference at the personal or group level.
4. c refers to the regression coefficients of personal biospheric values or group biospheric values on *environmental purchasing preference* at the personal level or the group level respectively.
5. c’ refers to regression coefficients between of personal biospheric values or group biospheric values on *environmental purchasing preference* at the personal level or the group level, controlling for environmental self- or group identity respectively.
6. *** *p* < .001, ** *p* < .01, * *p* <.05.

# References

De Groot, J. I. M., & Steg, L. (2007). Value Orientations and Environmental Beliefs in Five Countries. *Journal of Cross-Cultural Psychology*, *38*(3), 318–332. https://doi.org/10.1177/0022022107300278

Stuive, I., Kiers, H. A. L., & Timmerman, M. E. (2009). Comparison of methods for adjusting incorrect assignments of items to subtests: Oblique Multiple Group Method versus Confirmatory Common Factor Method. *Educational and Psychological Measurement*, *69*(6), 948–965. https://doi.org/10.1177/0013164409332226

Van der Werff, E., Steg, L., & Keizer, K. (2014). Follow the signal : When past pro-environmental actions signal who you are. *Journal of Environmental Psychology*, *40*, 273–282. https://doi.org/10.1016/j.jenvp.2014.07.004
